# ActRII blockade protects mice from cancer cachexia and prolongs survival in the presence of anti-cancer treatments

**DOI:** 10.1186/s13395-016-0098-2

**Published:** 2016-07-26

**Authors:** Shinji Hatakeyama, Serge Summermatter, Marie Jourdain, Stefan Melly, Giulia C. Minetti, Estelle Lach-Trifilieff

**Affiliations:** MusculoSkeletal Diseases, Novartis Institutes for Biomedical Research, Novartis Pharma AG, CH-4002 Basel, Switzerland

**Keywords:** ActRII blockade, Cancer cachexia, Combination, Cisplatin, Everolimus

## Abstract

**Background:**

Cachexia affects the majority of patients with advanced cancer and is associated with reduced treatment tolerance, response to therapy, quality of life, and life expectancy. Cachectic patients with advanced cancer often receive anti-cancer therapies against their specific cancer type as a standard of care, and whether specific ActRII inhibition is efficacious when combined with anti-cancer agents has not been elucidated yet.

**Methods:**

In this study, we evaluated interactions between ActRII blockade and anti-cancer agents in CT-26 mouse colon cancer-induced cachexia model. CDD866 (murinized version of bimagrumab) is a neutralizing antibody against the activin receptor type II (ActRII) preventing binding of ligands such as myostatin and activin A, which are involved in cancer cachexia. CDD866 was evaluated in association with cisplatin as a standard cytotoxic agent or with everolimus, a molecular-targeted agent against mammalian target of rapamycin (mTOR). In the early studies, the treatment effect on cachexia was investigated, and in the additional studies, the treatment effect on progression of cancer and the associated cachexia was evaluated using body weight loss or tumor volume as interruption criteria.

**Results:**

Cisplatin accelerated body weight loss and tended to exacerbate skeletal muscle loss in cachectic animals, likely due to some toxicity of this anti-cancer agent. Administration of CDD866 alone or in combination with cisplatin protected from skeletal muscle weight loss compared to animals receiving only cisplatin, corroborating that ActRII inhibition remains fully efficacious under cisplatin treatment. In contrast, everolimus treatment alone significantly protected the tumor-bearing mice against skeletal muscle weight loss caused by CT-26 tumor. CDD866 not only remains efficacious in the presence of everolimus but also showed a non-significant trend for an additive effect on reversing skeletal muscle weight loss. Importantly, both combination therapies slowed down time-to-progression.

**Conclusions:**

Anti-ActRII blockade is an effective intervention against cancer cachexia providing benefit even in the presence of anti-cancer therapies. Co-treatment comprising chemotherapies and ActRII inhibitors might constitute a promising new approach to alleviate chemotherapy- and cancer-related wasting conditions and extend survival rates in cachectic cancer patients.

**Electronic supplementary material:**

The online version of this article (doi:10.1186/s13395-016-0098-2) contains supplementary material, which is available to authorized users.

## Background

Cachexia affects the majority of patients with advanced cancer and is associated with a poor outcome, a reduction in treatment tolerance, response to therapy, quality of life, and survival rate. Skeletal muscle loss appears to be the most prominent event in cancer cachexia and cannot be fully reversed by conventional nutritional support [1, 2]. Recently, it has been shown in mouse models of ectopic lung and colon cancer that direct myostatin inhibition with a monoclonal antibody as well as indirect inhibition using a soluble ActRIIB-Fc protects from muscle wasting and even extends survival [3–6].

Several members of the transforming growth factor beta (TGF-β) superfamily, including myostatin, activin A, and growth differentiation factor 11 (GDF-11), are known to negatively regulate skeletal muscle mass in animals and humans throughout the lifecycle. The mechanism of myostatin signaling is complex due to activation of several downstream pathways [7]. Myostatin, activin, and GDF-11 bind to activin type II receptors (ActRIIA or ActRIIB) and induce their assembly with type I receptors. The absence of myostatin in developing animals and humans results in a hyper-muscular phenotype with an increased number and size of muscle fibers [8, 9]. Similarly, inhibition of myostatin action in adult animals increases muscle mass, suggesting that myostatin also restrains skeletal muscle mass in adulthood [10–12]. In contrast, high levels of myostatin or activin A have been reported to promote cachexia and the related muscle wasting in mice [13, 14]. Additionally, elevated circulating levels of activin A have clearly been correlated with the presence of cachexia in cancer patients [15, 16].

Bimagrumab is a human monoclonal antibody developed to bind competitively to ActRII with greater affinity than its natural ligands myostatin and activin A. It induces skeletal muscle hypertrophy and protects from dexamethasone-induced atrophy in mice [17] and is shown to improve the disease condition in the patients suffering from sporadic inclusion body myositis without causing serious adverse events [18]. Although it has been shown that the pharmacological blockade of ActRIIB ligands using a soluble receptor antagonist protects from cancer-induced cachexia in mice [4, 6], the effect of direct inhibition at the receptor level using an antibody approach has not been explored. In addition, cachectic patients with advanced cancer will receive anti-cancer as a standard of care, and whether specific ActRII inhibition remains efficacious when combined with anti-cancer agents has not been elucidated yet.

In this report, the effect of a chimeric mouse version of bimagrumab (CDD866) [17], which retains the binding, selectivity, and potency profile of bimagrumab while reducing risk for immunogenicity and enabling long-term profiling studies in mice, was evaluated in a CT-26 mouse colon cancer cachexia model to clarify interactions between CDD866 and various chemotherapies. Additionally, intervention at the activin type II receptors level via the use of the neutralizing Ab CDD866 is effective at protecting from cancer-induced cachexia as reported earlier through the blockade of circulating ligands (anti myostatin Ab or soluble ActRIIB-Fc).

Platinum-based drugs, such as cisplatin, are cytotoxic, intercalating agents that prevent DNA replication in a very unspecific manner and which are typically used as first-line therapy. Problematically, cisplatin has been shown to precipitate body and muscle weight loss as a side effect. We thus first aimed at evaluating the potential of CDD866 in countering cisplatin-mediated effects on muscle wasting. In a follow-up study, we assessed the impact of a more frequent dose of CDD866 and everolimus, a new generation, less cytotoxic, molecular-targeted agent, which inhibits the mammalian target of rapamycin (mTOR), on cancer cachexia.

## Methods

### Reagents

Bimagrumab is a human, monoclonal antibody directed against ActRII and originally identified from the MorphoSys HuCal phage library. CDD866 is a murinized version of bimagrumab, where the human Fc region of the antibody has been replaced by a mouse Fc. CDD866 was produced in CHO cells at Novartis Pharma AG (Basel, Switzerland). Cisplatin (cis-diamminedichloro-platinum (II)) was purchased from Sigma Aldrich (catalog number 479306). Everolimus was synthesized at Novartis Pharma AG.

### Animal experiments

Studies described in this report were performed according to the regulations effective in the Canton of Basel-City, Switzerland, under the license number BS-2186. Adult male Balb/cJRj mice at the age of 11 to 12 weeks were purchased from Janvier Laboratories (Le Genest St Isle, France). Mice were acclimated to the facility for 7 days. Animals were housed in groups of five or less animals at 25 °C with a 12:12 h light-dark cycle. They were fed a standard laboratory diet containing 18.2 % protein and 3.0 % fat with an energy content of 15.8 MJ/kg (NAFAG 3890, Kliba, Basel, Switzerland). Food and water were provided ad libitum.

Mouse colon cancer cell line CT-26 was obtained from Dr. Chatenay-Rivauday at Novartis Pharma AG (Basel, Switzerland) and cultured in RPMI 1640 medium supplemented with 10 % heat inactivated fetal bovine serum and antibiotic-antimycotic solution at 37 °C with 5 % CO_2_. CT-26 cells were harvested by treatment with Accutase® (PAA Laboratories GmbH, Pasching, Austria) and suspended in a solution containing 50 % PBS and 50 % BD Matrigel™ Matrix without phenol red (catalog number 356237, BD Biosciences, Bedford, MA, USA). A 0.1 mL of cell suspension containing 3 × 10^5^ cells was inoculated subcutaneously into the left flank of mice. When tumors were palpable (e.g., 60 mm^3^), mice bearing tumors were randomized to produce groups balanced with respect to mean and range of tumor sizes and body weight. Treatments were initiated on the day of randomization, which is referred to as day 0. Importantly, muscle wasting was apparent at the time-point of treatment initiation as assessed by MRI of the calf muscles (Additional file 1: Figure S1).

Therapeutic intervention studies were conducted to evaluate the effect of CDD866, either alone or in combination with anti-cancer agents. CDD866 was administered at 20 mg/kg s.c., once weekly for combination with cisplatin or twice weekly for combination with everolimus in a volume of 5 mL/kg, and the last administration occurred the day before necropsy. Cisplatin was administered at 1 mg/kg i.p. twice a week. A dose of 1 mg/kg had been selected to avoid excessive body weight loss, while maintaining a significant anti-tumor effect. Everolimus was administered at 5 mg/kg p.o. once daily, and the last administration was a few hours before necropsy. In the combination groups, cisplatin or everolimus treatment was combined with once or twice weekly subcutaneous treatment of CDD866, respectively. Body weight and tumor volume were measured two to three times per week. At the end of the experiment, the mice were euthanized with CO_2_, and tumor, tibialis anterior, gastrocnemius-soleus-plantaris complex, and quadriceps were collected and weighed.

Time-to-progression studies were performed as a follow-up to assess if the combination of CDD866 and cisplatin or everolimus slows progression of cancer cachexia in respect to interruption criteria which were defined by body weight loss reaching 20 % or tumor volume exceeding 1500 mm^3^. The treatment regimens were identical to those used in the therapeutic intervention studies. Body weight and tumor volume were measured two to three times per week in the first 2 weeks and then every day until the end of experiment. The mice were euthanized with CO_2_, when body weight loss was close to 20 % or tumor volume exceeded 1500 mm^3^.

### Protein analysis

Lysis buffer consisting of extraction reagent (Phosphosafe; Novagen Inc., Madison, WI, USA) supplemented with 1 % protease inhibitor cocktail (calbiochem# 539131) and 0.2 % SDS was added to frozen muscles and homogenized using a Precellys FastPrep-Machine FP20. Homogenates were separated by centrifugation for 20 min at 4 °C (14,000 rpm). Supernatants were collected and protein contents measured a commercial kit for protein determination (BCA Kit; Thermo Scientific). Samples were diluted in SDS-PAGE sample buffer and denatured for 10 min at 70 °C. Equal amounts of protein were loaded per lane of 4 to 12 % and 8 % polyacrylamide gel (NuPAGE Bis-Tris gel; Invitrogen Corp., Carlsbad, CA, USA), separated by electrophoresis, and then transferred onto nitrocellulose membranes. Membranes were blocked in TBS with 0.1 % Tween and 5 % *w*/*v* non-fat milk powder. Primary antibodies phospho-SMAD3 (Millipore #04 1042 diluted 1:1000) and α-tubulin (Sigma T6199 Diluted 1:5000) were incubated in TBS with 0.1 % Tween 20 and 5 % *w*/*v* non-fat milk powder and secondary antibodies in TBS with 0.1 % Tween 20, 0.05 % SDS, and 5 % non-fat milk. Immunoreactivity was detected by SuperSignal West Femto Maximum Sensitivity Substrate (Thermo Scientific) and exposed to film or acquired by FusionSpectra. Quantitative determination of mTOR and IL-6 was performed using an assay kit (catalog number K15170D for phospho (Ser 2448)/total mTOR, K15048D for IL-6) from MesoScale Discovery using a MesoScale Discovery reader according to the manufacturer’s instruction.

### Gene expression profiling

RNA samples were extracted from the gastrocnemius muscle using the Trizol reagent (Invitrogen). Reverse transcription was performed with random hexamers on 1 μg of total RNA using a high-capacity reverse transcription kit (Applied Biosystems), and the reaction mixture was diluted 100 times for amplification. PCRs were performed in duplicates in 384-well plates on a CFX384 cycler (Bio-Rad, Hercules, CA, USA) using specific TaqMan probes (Applied Biosystems). Data were normalized to two housekeeping genes using the ΔΔCT threshold cycle (CT) method.

### Statistical analysis

Values are expressed as mean ± SEM. Statistical analysis was carried out using Holm-Sidak’s multiple comparison test following analysis of variance to compare the treatment groups to the control groups (non-tumor and tumor-bearing), anti-cancer agent alone (cisplatin or everolimus) or CDD866 alone in the therapeutic intervention study, and Dunn’s multiple comparisons test for time-to-progression study. Differences were considered to be significant when the probability value was <0.05. Statistical analyses were performed by GraphPad Prism (GraphPad Software, Inc., La Jolla, CA, USA). Body weight was expressed as percentage change from day 0 as the start of treatment. Tumor volumes in cubic millimeters were calculated according to the formula (length × width^2^)/2. Muscle weight was normalized to the body weight on the day of cell inoculation (initial body weight) and then expressed as percentage change from the non-tumor control group.

## Results

Cancer cachexia, i.e., muscle wasting associated with cancer and also with some standard of care interventions, dramatically affects patient quality of life, anti-cancer treatment effectiveness, and overall survival. We characterized our anti-cachexia agent, CDD866, and examined its potential benefit in the context of co-therapies in CT-26 mouse colon cancer cachexia model, in which tumor is insensitive to anti-ActRII intervention. Chemotherapy constitutes a standard of care in many cancers and is frequently used as first-line therapy. Intriguingly, certain chemotherapeutic agents, which are routinely administered to hinder tumor growth, precipitate muscle wasting. Indeed, administration of cisplatin is known to exacerbate body weight and muscle loss in mouse cancer cachexia. We thus first evaluated whether CDD866 could counter cisplatin-induced wasting without affecting the efficacy of the chemotherapy.

### CDD866 prevents cisplatin-induced body weight loss

**Fig. 1:**
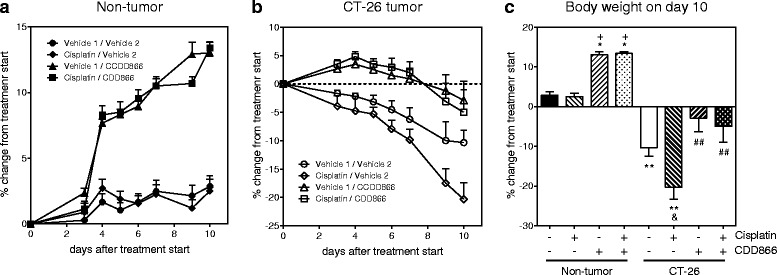
Effects of cisplatin and CDD866 on body weight. Non-tumor (**a**), CT-26 tumor (**b**), and body weight on day 10 (**c**). Values are expressed as means ± SEM (*n* = 7–8). Percent changes of body weight were calculated in comparison to treatment start. ^*^ *P* < 0.05, ^**^ *P* < 0.01 versus non-tumor control; ^++^ *P* < 0.01 versus non-tumor cisplatin; ^&^ *P* < 0.05 versus CT-26 control; ^##^ *P* < 0.01 versus CT-26 cisplatin

Extensive body weight loss has emerged as a key determinant of cancer-related death. We thus longitudinally monitored body weight development in non-tumor and tumor bearing mice (Fig. 1a, b). Ten days after starting the treatment, tumor-bearing animals receiving cisplatin as a mono-therapy had lost 20 % of their initial body weight (Fig. 1b, c). By contrast, vehicle-treated, tumor-bearing animals experienced a body weight decrease of 10 %, while animals treated with CDD866 alone or in combination with cisplatin exhibited moderate body weight losses of only 3 and 5 %, respectively (Fig. 1b, c). In healthy control animals, cisplatin did not affect body weight and CDD866 administration resulted in a marked body weight gain in the absence and presence of cisplatin (Fig. 1a, c). These data demonstrate that cisplatin, at an effective anti-tumor dose (cf. also paragraph below), indeed precipitates body weight loss in cachectic animals and that CDD866 significantly reduces chemotherapy-induced wasting as well as reducing the cancer-induced body weight loss.

**Fig. 2:**
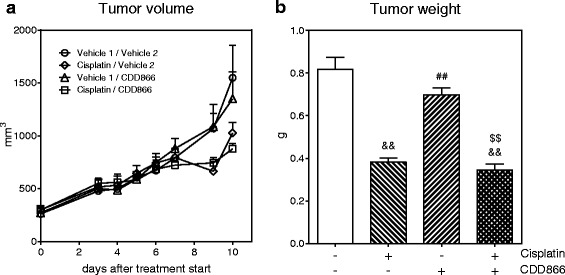
Effects of cisplatin and CDD866 on tumor. Tumor volume (**a**) and tumor weight (**b**). Values are expressed as means ± SEM (*n* = 6–8). ^&&^ *P* < 0.01 versus CT-26 control; ^##^ *P* < 0.01 versus CT-26 cisplatin; ^$$^ *P* < 0.01 versus CT-26 CDD866

Major concerns to be addressed in this study were potential drug-drug interactions and specifically whether CDD866 might reduce the efficacy of chemotherapy and therefore impact tumor growth promotion. At treatment initiation, the average tumor volume was ≥260 mm^3^ (Fig. 2a). CDD866 neither accelerated tumor progression (Fig. 2a, b) nor did it impair the anti-tumor effect of cisplatin (Fig. 2a, b). Thus, CDD866 is efficacious in reducing chemotherapy-mediated body weight loss in cancer cachexia without interfering with the anti-tumor effect of cisplatin.

### CDD866 antagonizes cisplatin-induced muscle wasting

**Fig. 3:**
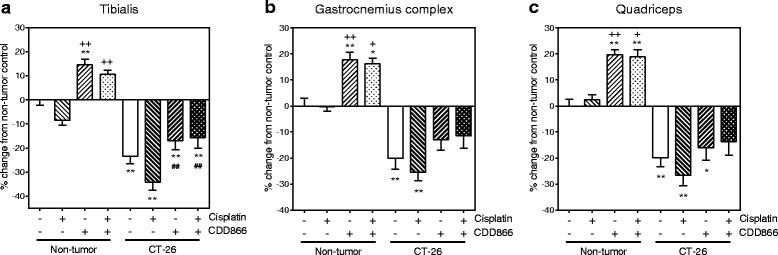
Effects of cisplatin and CDD866 on muscle weight. Tibialis (**a**), gastrocnemius complex (**b**), and quadriceps (**c**). Values are expressed as means ± SEM (*n* = 6–8). Percent changes of muscle weight, normalized to initial body weight, were calculated in comparison to non-tumor control. ^*^ *P* < 0.05, ^**^ *P* < 0.01 versus non-tumor control; ^+^ *P* < 0.05, ^++^ *P* < 0.01 versus non-tumor cisplatin; ^##^ *P* < 0.01 versus CT-26 cisplatin

Given the positive effect of CDD866 on body weight, we next determined the impact of the various interventions on individual skeletal muscles. In gastrocnemius-soleus-plantaris complex, cisplatin exacerbated muscle weight loss up to 25 % compared to 20 % for tumor alone. CDD866 treatment tended to reduce muscle weight loss to 13 % and this protective effect was preserved in the presence of cisplatin (12 %) (Fig. 3b). A similar level of protection was observed in quadriceps muscle (Fig. 3c). Tibialis anterior benefited most from CDD866 treatment. In tibialis anterior, cisplatin-treated animals experienced a muscle wasting of 34 % and co-administration of CDD866 reduced muscle loss significantly to 16 % (Fig. 3a).

### CDD866 in combination with cisplatin delays time-to-progression

Extensive tumor growth and subsequent body weight loss are important predictors of mortality in cancer patients. We therefore wanted to evaluate whether the combination of CDD866 and cisplatin has an impact on the length of survival. For ethical reason, we abstained from classical survival studies. Instead, each mouse was individually euthanized when experiencing either a body weight loss exceeding 20 % of initial body weight or reaching a tumor volume of 1500 mm^3^, which is referred to as time-to-progression.

**Fig. 4:**
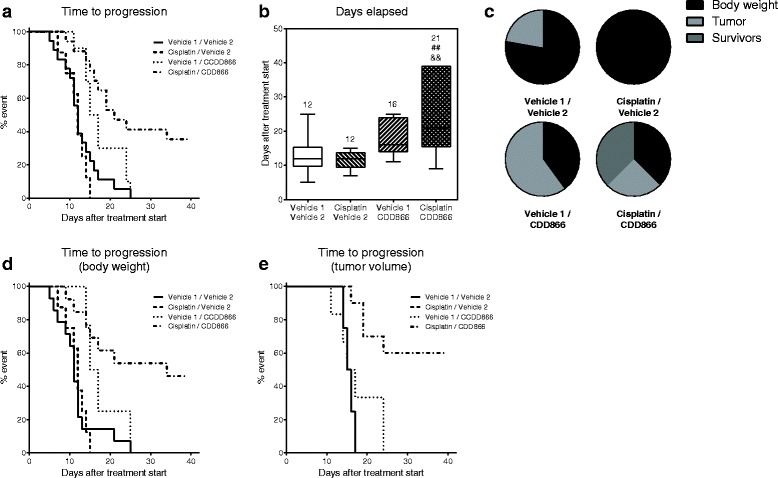
Effects of cisplatin and CDD866 on time-to-progression. Time-to-progression expressed by percentage of event defined by interruption criteria (**a**); median days elapsed before reaching an interruption criterion (**b**), expressed by box and whiskers with min to max; ^&&^ *P* < 0.01 versus CT-26 control; ^##^ *P* < 0.01 versus CT-26 cisplatin. Distribution by termination criterion (**c**) and breakdown of time-to-progression by body weight loss (**d**) and tumor volume (**e**)

On average, animals receiving vehicle or cisplatin had to be sacrificed after 12 days (Fig. 4a, b). CDD866-treated animals had to be euthanized after 16 days, which corroborates previous findings that CDD866 treatment reduced body weight loss but did not promote tumor growth. The combined treatment of CDD866 and cisplatin was superior to any other intervention tested. Indeed, combination treatment extended time-to-progression up to 21 days (Fig. 4b). Monitoring was stopped after 39 days with 35 % of animals in the combination group still not having reached one of the defined interruption criteria (Fig. 4a).

Additional partitioning analyses showed that animals treated with cisplatin were exclusively euthanized due to body weight loss, while co-treatment with CDD866 substantially reduced dropout based on body weight loss (Fig. 4c–e).

### CDD866 and everolimus prevent cancer cachexia in an additive way

In the next step, we selected everolimus, a new generation molecular-targeted agent against mammalian target of rapamycin (mTOR), as a combination partner because mTOR is known to play a pivotal role in cell growth and proliferation. In addition, treatment frequency for CDD866 was increased to twice weekly to ensure significant anti-cachectic effect when administered as single agent, and the combination of everolimus and CDD866 was evaluated in non-tumor mice as well as tumor-bearing cachectic mice.

**Fig. 5:**
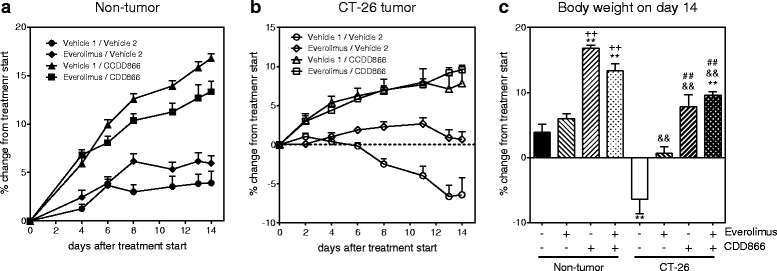
Effects of everolimus and CDD866 on body weight. Non-tumor (**a**), CT-26 tumor (**b**), and body weight on day 14 (**c**). Values are expressed as means ± SEM (*n* = 8–10). Percent changes of body weight were calculated in comparison to treatment start. ^**^ *P* < 0.01 versus non-tumor control; ^++^ *P* < 0.01 versus non-tumor everolimus; ^&&^ *P* < 0.01 versus CT-26 control; ^##^ *P* < 0.01 versus CT-26 everolimus

In the non-tumor bearing group, body weight gain was not affected significantly by everolimus treatment. In contrast, body weight gain increased significantly with CDD866 treatment as expected (Fig. 5a, c). The effects of CDD866 on body weight gain could not be attributed to alterations in food intake. The body weight increase was slightly lower in the combination group (Fig. 5a, c) but still significantly different from everolimus alone and not significantly different from CDD866 alone up to the termination on day 14. In the CT-26 group, body weight was significantly decreased in the tumor-bearing control group on day 14 when compared to the non-tumor control group (Fig. 5b, c). CT-26-induced loss in body weight was completely prevented by everolimus, CDD866 and the combination of everolimus and CDD866. The effect of CDD866 on body weight was maintained in the presence of everolimus.

**Fig. 6:**
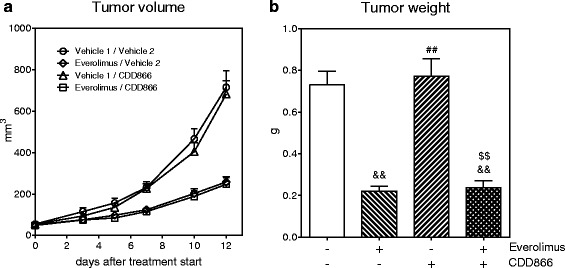
Effects of everolimus and CDD866 on tumor. Tumor volume (**a**) and tumor weight (**b**). Values are expressed as means ± SEM (*n* = 10). ^&&^ *P* < 0.01 versus CT-26 control; ^##^:*P* < 0.01 versus CT-26 everolimus; ^$$^: *P* < 0.01 versus CT-26 CDD866

Everolimus slowed CT-26 tumor growth, and the anti-tumor effect was maintained in the presence of CDD866 (Fig. 6a). CT-26 tumor weight was significantly reduced with everolimus treatment alone or in combination with CDD866 (Fig. 6b). There was no significant effect of CDD866 treatment on CT-26 tumor weight (Fig. 6b).

**Fig. 7:**
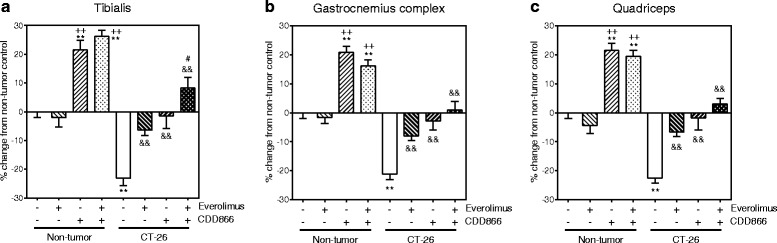
Effects of everolimus and CDD866 on muscle weight. Tibialis (**a**), gastrocnemius complex (**b**), and quadriceps (**c**). Values are expressed as means ± SEM (*n* = 10). Percent changes of muscle weight, normalized to initial body weight, were calculated in comparison to non-tumor control. ^**^ *P* < 0.01 versus non-tumor control; ^++^ *P* < 0.01 versus Non-tumor everolimus; ^&&^ *P* < 0.01 versus CT-26 control; ^#^ *P* < 0.05 versus CT-26 everolimus

In the non-tumor bearing group, the weight of tibialis anterior, gastrocnemius-soleus-plantaris complex and quadriceps muscles was not affected by everolimus treatment and significantly increased by CDD866 treatment (Fig. 7a–c). The effect of CDD866 on muscle weight was maintained in the presence of everolimus. CT-26 tumor induced a significant decrease in the weight of tibialis anterior, gastrocnemius-soleus-plantaris complex, and quadriceps muscles compared to the non-tumor bearing control group (Fig. 7a–c). CT-26-induced muscle weight loss was significantly reduced by everolimus or CDD866 treatment. Interestingly, the combination of everolimus and CDD866 appeared to reverse skeletal muscle weight loss in an additive manner, and this combined treatment effect was significantly different from the everolimus treatment alone.

### CDD866 in combination with everolimus delays time-to-progression

**Fig. 8:**
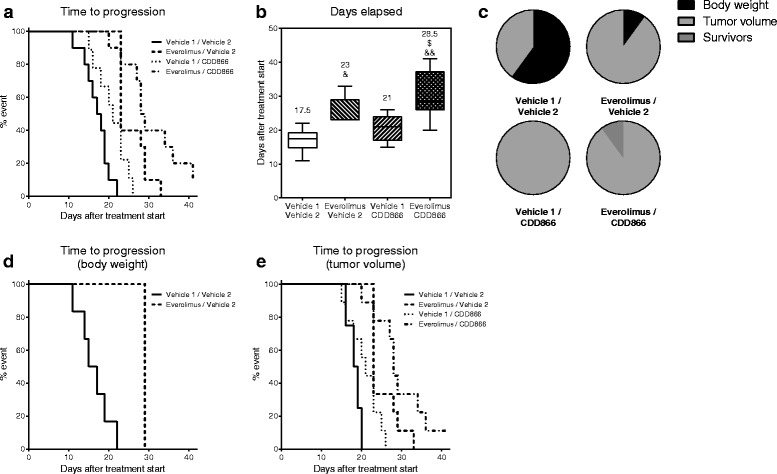
Effects of everolimus and CDD866 on time-to-progression. Time-to-progression expressed by percentage of event defined by interruption criteria (**a**); median days elapsed before reaching an interruption criterion (**b**), expressed by box and whiskers with min to max; ^&^ *P* < 0.05, ^&&^ *P* < 0.01 versus CT-26 control; ^$^ *P* < 0.05 versus CT-26 CDD866. Distribution by termination criterion (**c**) and breakdown of time-to-progression by body weight loss (**d**) and tumor volume (**e**)

In addition to the beneficial effects of everolimus and CDD866 on CT-26-induced cachexia in the therapeutic intervention study, the effect of these treatments on progression of cancer and the associated cachexia was evaluated, using the same criteria as in the prior cisplatin combination study. In the CT-26 control group, the median days elapsed until an interruption criterion (time-to-progression) was 17.5 days after randomization and treatment start (Fig. 8a, b). Everolimus treatment significantly prolonged time-to-progression to 23 days mainly due to its anti-tumor effect, while CDD866 showed only a non-significant trend of extension to 21 days. The lack of significance of CDD866 on time-to-progression is explained by the fact that, although the treatment was highly successful in preventing body weight loss, it did not inhibit tumor growth, which was the second interruption criterion. Importantly, the combination of everolimus and CDD866 appeared to further slow time-to-progression to 28.5 days, an effect which was significant compared to the CT-26 control group.

When the distribution of interruption criteria was compared among groups, tumor volume was applied in the treatment groups in all cases with an exception of one case in everolimus treatment alone and one survivor in the combination group, while body weight contributed to more than half of termination in the CT-26 control group (Fig. 8c–e).

### CDD866 and everolimus modulate ActRII and mTOR signaling pathways

**Fig. 9:**
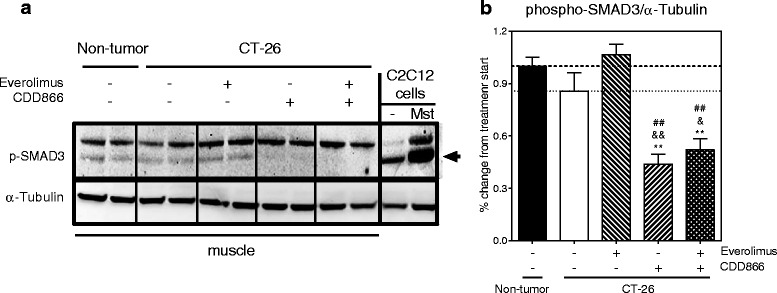
Effects of everolimus and CDD866 on phosphorylation of SMAD3 levels. Western blot image (**a**) and quantified value (**b**). Differentiated C2C12 cells stimulated with or without myostatin (Mst) were used as a positive control for detection of phosphorylation of SMAD3 levels. The lower, weaker band represents phosphorylated SMAD3. Values are expressed as means ± SEM (*n* = 5–10); **P < 0.01 versus non-tumor control; ^&^ *P* < 0.05, ^&&^ *P* < 0.01 versus CT-26 control; ^##^ *P* < 0.01 versus CT-26 everolimus

**Fig. 10:**
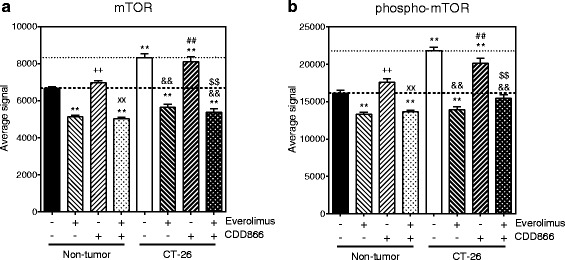
Effects of everolimus and CDD866 on phosphorylation of mTOR levels. Total mTOR (**a**) and phospho-mTOR (**b**). Animals received their last injection of CDD866 on day 13 and were euthanized on day 14. Values are expressed as means ± SEM (*n* = 5–10); ^**^ *P* < 0.01 versus non-tumor control; ^++^ *P* < 0.01 versus non-tumor everolimus; ^xx^ *P* < 0.01 versus non-tumor CDD866; ^&&^ *P* < 0.01 versus CT-26 control; ^##^ *P* < 0.01 versus CT-26 everolimus; ^$$^ *P* < 0.01 versus CT-26 CDD866

**Fig. 11:**
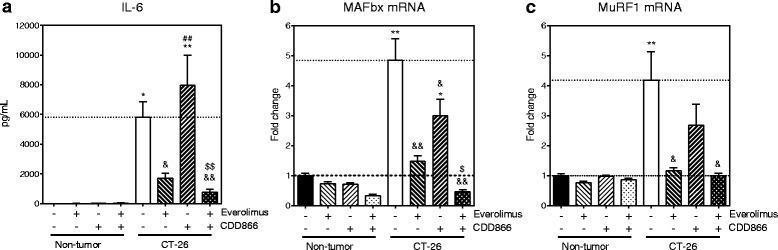
Effects of everolimus and CDD866 on inflammation and atrophy markers. IL-6 (**a**), MAFbx (**b**), and MuRF1 (**c**) levels. Animals received their last injection of CDD866 on day 13 and were euthanized on day 14. Values are expressed as means ± SEM; ^*^ *P* < 0.05, ^**^ *P* < 0.01 versus non-tumor control; ^&^ *P* < 0.05, ^&&^ *P* < 0.01 versus CT-26 control; ^##^ *P* < 0.01 versus CT-26 everolimus; ^$$^ *P* < 0.01 versus CT-26 CDD866

Since the combination of everolimus and CDD866 appeared to be the most efficacious treatment, selected key signaling events were examined. As expected with ActRII blockade with CDD866, phosphorylation of SMAD3 was significantly reduced in the CDD866 treatment alone and in combination with everolimus (Fig. 9a, b). Total protein levels and phosphorylation of mTOR was significantly inhibited in the everolimus treatment alone and in combination with CDD866, as an indicator of everolimus inhibition through S6K (Fig. 10a, b). Furthermore, inflammation status was evaluated by analyzing levels of inflammatory cytokine IL-6 which are known to be increased in the CT-26-induced cachexia conditions. IL-6 was significantly increased in the tumor-bearing control, and the everolimus treatment alone or in combination with CDD866 significantly reduced IL-6, but the CDD866 treatment alone did not show a significant effect on IL-6 (Fig. 11a). Moreover, in CT-26 tumor bearing mice, messenger RNA (mRNA) levels of atrogenes, MAFbx, and MuRF1 were significantly up-regulated in skeletal muscles, and those increases were significantly inhibited by everolimus or by the combination with CDD866 while CDD866 only displayed a trend towards reducing MuRF1 (Fig. 11b, c). CDD866 treatment inhibited MAFbx up-regulation significantly but partially, although everolimus or CDD866 treatment significantly reduced CT-26-induced muscle weight loss.

## Discussion

The ActRII neutralizing antibody CDD866 and anti-cancer agents, cisplatin and everolimus, were evaluated when administered alone or in combination in a model of CT-26 mouse colon cancer-induced cachexia, to assess whether CDD866 remains efficacious upon co-administration and whether such a combination is superior to the respective mono-therapy in delaying disease progression. All therapeutic interventions were initiated after the onset of muscle wasting.

Despite substantial tumor growth inhibition, cisplatin accelerated body weight loss in cachectic animals, likely due to the high toxicity of the anti-cancer agent. CDD866 fully prevented cisplatin-mediated body weight loss demonstrating that ActRII inhibition remained efficacious in the presence of cisplatin. Cisplatin treatment alone and also in combination with CDD866 reduced CT-26 tumor weight to similar levels, which underlines that the anti-cancer effect of cisplatin was not negatively affected by CDD866.

Consistently, cisplatin treatment did not improve CT-26 tumor-induced skeletal muscle wasting but rather tended to exacerbate skeletal muscle loss. In contrast, administration of CDD866 alone or in combination with cisplatin protected from skeletal muscle weight loss compared to animals receiving only cisplatin, corroborating further that ActRII inhibition remains fully efficacious under cisplatin treatment. These results thus demonstrate that CDD866 in combination with cisplatin counters muscle wasting in cachectic animals when compared to cisplatin treatment alone. Noteworthy, CDD866 was administered only once per week and mice received only two injections throughout the entire study (apart from the survival studies). Since elevated release of activin A has been reported from cancer tissues [19] and associated with cancer cachexia phenotype in patients [15], a higher dosing or frequency of dosing of CDD866 might be required in cancer cachexia models to elicit more pronounced or maximal responses. Indeed, stronger muscle wasting sparing was noticed with CDD866 alone in the combination study with everolimus under a more frequent dosing regimen.

Cancer patients with low muscle mass are at increased risk for treatment-related toxicities from chemotherapy and show increased overall mortality [20]. Consistently, CDD866 significantly delayed disease progression largely by increasing muscle mass. Time-to-progression in cancer cachexia was even further retarded by concomitant therapy with CDD866 and cisplatin, which simultaneously countered muscle wasting and inhibited tumor growth.

Since mTOR is known to play a pivotal role in cell growth and proliferation, mTOR inhibition by everolimus exhibited significant anti-tumor effect as expected, both in the absence and presence of CDD866. This result clearly shows that anti-cancer effect of everolimus is not affected negatively by ActRII inhibition with CDD866. In line with body weight decreases caused by CT-26 tumor, skeletal muscle weight was significantly decreased in the CT-26 control group. Everolimus or CDD866 treatment alone significantly protected the tumor-bearing mice against skeletal muscle weight loss caused by CT-26 tumor. Interestingly, ActRII inhibition by CDD866 not only remained efficacious in the presence of everolimus but also showed a non-significant trend for an additive effect at reversing skeletal muscle weight loss, despite the fact that mTOR is required for normal muscle growth [21, 22]. Similarly, in the non-tumor-bearing mice, there was no effect on body weight upon everolimus treatment, while CDD866 increased body weight significantly. The effect of CDD866 on body weight was maintained in the presence of everolimus, clearly showing that the mTOR inhibition did not alter the effect of CDD866 on body weight. Also, the muscle anabolic response observed upon CDD866 treatment in non-tumor bearing mice was significant and not affected by mTOR inhibition at dose clearly effective on tumor.

Everolimus treatment alone prolonged time-to-progression as a surrogate for survival and also CDD866 showed a trend of extension. Importantly, the combination of everolimus and CDD866 appeared to further slow-down time-to-progression. Each treatment worked complementary to exert a beneficial effect, with everolimus inhibiting tumor growth and CDD866 preventing cachexia. A trend for an additive anti-cachectic effect observed in the combination of CDD866 and everolimus would warrant further exploration as to how ActRII blockade and mTOR inhibition may positively interact on skeletal muscle undergoing cachexia.

It is reported that mTORC1 is activated in denervation-induced skeletal muscle atrophy [23–26] and further downstream activation of MAFbx and MuRF1 to promote atrophy, but the anti-atrophy effect of mTOR inhibition by rapamycin treatment was inconclusive [25, 26]. Activation of mTOR is also reported in other pathological conditions, such as aging [27], obesity, insulin resistance, and diabetes [28], where mTOR inhibition seems to be beneficial [29]. In the present study, there was a significant increase in phosphorylation as well as the total amount of mTOR in skeletal muscles of tumor-bearing mice. Therefore, the everolimus benefit observed at countering muscle wasting may result for some degree from a direct mTOR inhibition at the muscle level, while another part may come from its anti-tumor efficacy. In addition, although everolimus alone reduced MAFbx and MuRF1 down to baseline, an additional anti-cachexia benefit could be observed when combined with ActRII blockade.

## Conclusions

Collectively, our studies thus demonstrate that blocking ActRII with CDD866, even after onset of muscle mass loss, partially reverses cancer—as well as cisplatin-induced wasting and clearly delays time-to-progression in cancer cachexia. CDD866 was also effective in preventing CT-26 tumor-induced systemic cachexia in the presence of everolimus. Importantly, there was no obvious deleterious interaction between CDD866 and anti-cancer agents such as cisplatin and everolimus. Together with data demonstrating that blockade of the myostatin/ActRIIB pathway is highly beneficial in models of cancer-induced cachexia [3–6], our results indicate that ActRII blockade might be beneficial in cachectic cancer patients which are or have been exposed to chemotherapies.

## Abbreviations

ActRII, activin receptor type II; GDF-11, growth differentiation factor 11; mTOR, mechanistic target of rapamycin; TGF-β, transforming growth factor beta

## Additional file

Additional file 1: Figure S1. Decreased calf muscle volume in CT-26 colon cancer-bearing mice. Calf muscle volume assessed non-invasively by MRI at the time-point of treatment initiation. Values are expressed as percentage change from basal ± SEM; ^**^ *P* < 0.01 versus non-tumor control. (PDF 28 kb)
