# Supplementary figures and images for: ActRII blockade protects mice from cancer cachexia and prolongs survival in the presence of anti-cancer treatments
Source: Skelet Muscle. 2016 Jul 26;6:26. doi: 10.1186/s13395-016-0098-2 (PMC4960708; doi:10.1186/s13395-016-0098-2)

# Supplimental figure 1

## Muscle volume change

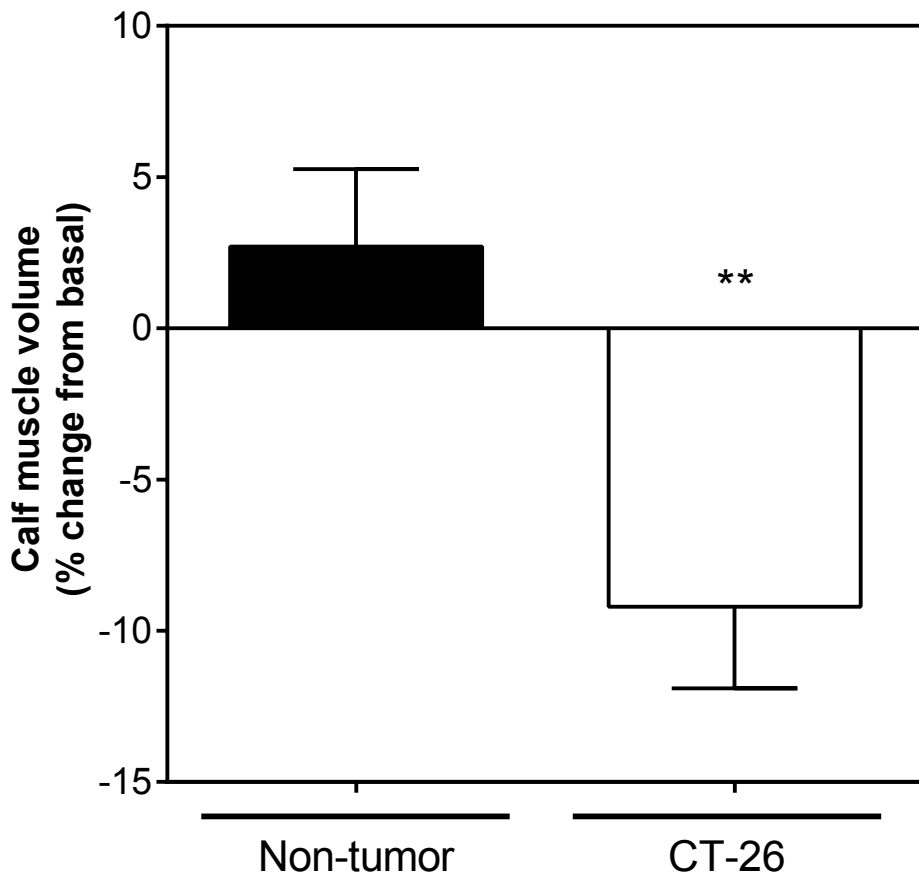

Supplement: Additional file 1: Figure S1. — Decreased calf muscle volume in CT-26 colon cancer-bearing mice. Calf muscle volume assessed non-invasively by MRI at the time-point of treatment initiation. Values are expressed as percentage change from basal ± SEM; ** P < 0.01 versus non-tumor control. (PDF 28 kb) [file 13395_2016_98_MOESM1_ESM.pdf]
